# A novel pipeline for realistic synthetic longitudinal EHR data generation

**DOI:** 10.21203/rs.3.rs-8497559/v1

**Published:** 2026-01-29

**Authors:** Gabrielle Josling, Ibrahima Diouf, Sankalp Khanna

**Affiliations:** Commonwealth Scientific and Industrial Research Organisation

**Keywords:** Synthetic data, electronic health records, synthetic data utility

## Abstract

**Background:**

Synthetic health data offers a promising means of sharing clinical information without compromising patient privacy. However, existing methods often produce outputs that differ in structure from real data and are evaluated in narrow contexts, limiting their practical use in downstream analytical workflows. This study introduces a pipeline that builds upon existing methods for generating realistic synthetic longitudinal electronic health record data, evaluates it across three diverse datasets, and offers evidence-based guidance on the use of synthetic data to replace or augment real data.

**Methods:**

The pipeline extends existing state of the art HALO and ConSequence frameworks with a post-processing step that reconstructs continuous variables and timestamps, producing synthetic data that closely matches the structure of real medical record datasets. It was applied to three clinically diverse datasets: a small longitudinal cohort, a medium-sized intensive-care dataset, and a very large multi-hospital administrative dataset. Realism was assessed alongside utility for machine learning, statistical modelling, and time series analysis tasks.

**Results:**

Across all datasets, the pipeline generated realistic synthetic data that preserved key statistical properties and relationships. Machine learning models trained on synthetic data achieved similar predictive accuracy and feature importance patterns to those trained on real data, indicating strong utility. Synthetic data also performed well in statistical modelling, with the direction and magnitude of effects generally closely aligned with the real data. However, it may be less suitable when precise estimates are required or when modelling relatively rare conditions. Importantly, although the pipeline reconstructed timestamp structures, it did not capture aggregate temporal patterns and the resulting data was therefore unsuitable for time series analysis.

**Conclusions:**

The pipeline produces realistic and analytically useful synthetic longitudinal electronic health record data across datasets of widely varying scales. These findings provide practical guidance on when synthetic data can meaningfully substitute for or complement real data.

## Background

Clinical and health research increasingly depends on rich, structured datasets such as electronic health records (EHRs), disease registries, and longitudinal cohort studies. These tabular datasets provide the foundation for epidemiological research, assessment of healthcare outcomes, and data-driven policy development. Yet despite their value, access to such data is often restricted due to privacy concerns. Synthetic tabular data has emerged as a promising way to expand access while protecting patient privacy, address biases in real-world datasets, and support analysis of rare diseases or under-represented groups where real data are limited [1, 2].

Tabular clinical data can be characterised into three main types: snapshot (or cross-sectional), longitudinal, and time series data [3]. Snapshot data consists of a single record per individual at a specific point in time. In contrast, longitudinal data involves multiple records per individual collected over time and at irregular intervals (such as repeated visits to hospital). Time series data instead typically originates from monitoring devices (e.g., EEGs) and has regular, evenly spaced timepoints. Although both longitudinal and time series data involve repeated measurements, their temporal structure and origins differ substantially.

Of these, longitudinal data is of particular relevance for health applications. However, generating realistic synthetic longitudinal data, such as EHRs, poses several challenges. These include:

- Relational structure and hierarchical dependencies: Clinical datasets often span multiple interrelated tables, such as patients, admissions, diagnoses, procedures, and laboratory results. These tables include both static attributes (such as demographic information) and time-varying information (such as repeated lab tests and multiple visits). The data is also inherently hierarchical, as a single patient may have multiple visits and each visit may involve multiple diagnoses and procedures. Maintaining consistency and coherence across these linked records is a significant challenge for synthetic data generation.
- Longitudinal nature: Clinical datasets can capture multiple encounters for the same individual over time. These events typically occur at irregular intervals and both the sequence and timing of events is meaningful. Because of this, synthetic data must be able to capture both sequential dependencies (such as the progression of diagnoses) and temporal dependencies (such as the interval between events) [3]. Accurately capturing this longitudinal structure is essential for many downstream tasks.
- Missing data: Clinical datasets often have a large number of missing values. Importantly, these values are frequently not missing at random but instead are clinically meaningful [4]. For example, the absence of a laboratory test result may indicate a clinician deemed that test unnecessary, so the missing value can convey information about the patient’s condition. In this context, missingness can be informative and thus should be captured in synthetic data. Despite this, few approaches explicitly model or evaluate the patterns of missingness in the data.
- High dimensionality: Clinical datasets are often characterised by a very large number of variables, including demographics, details of the encounter (e.g., visit type, triage category, arrival mode, provider, diagnoses, medications, and laboratory results) [1]. This makes modelling relationships between different variables very complex, particularly when there may be many missing values. High dimensionality also makes it more likely there are rare combinations of features that if reproduced in synthetic data may inadvertently reveal information about real individuals.
- Heterogeneous features: Clinical datasets include multiple variable types, including timestamps, categorical variables (e.g., gender, visit type), and continuous variables (e.g., length of stay, lab values). However, many synthetic data generation methods are specialised for either categorical or continuous variables and so can’t readily produce the full range of variables found in real data [3].
- Privacy: Clinical data is inherently sensitive and subject to strict privacy requirements, making it unsuitable for sharing widely. Although increased ability to share data is frequently cited as a benefit of synthetic data, in principle it’s possible for information about real individuals included in the data used to train a model to be inferred from synthetic data [5]. However, there is currently no widely accepted standard for assessing privacy risk in synthetic datasets.

The majority of methods for generating synthetic tabular data use deep learning, a type of machine learning that involves training neural networks to learn complex patterns in data [2]. This includes approaches such as autoencoders [6, 7], generalised adversarial models [8-11], diffusion models [12, 13], and even language models [14, 15].

Although many methods for generating synthetic clinical data have been described, none fully address all of the challenges listed above [16]. Many methods generate only snapshot data and therefore cannot be used for EHRs. Of those that can generate longitudinal data, many can only generate sequences of diagnostic codes across visits and do not capture the intervals between visits or other variables associated with each visit. Many methods can generate categorical or continuous data, but not both. This dramatically limits the utility of the resulting synthetic data for most applications.

The Hierarchical Autoregressive Language mOdel (HALO) and its rule-based extension ConSequence developed by Theodorou et al. address many of these limitations [15, 17]. HALO can encode both static patient demographic information and visit-level features, and also captures both sequential and temporal patterns in longitudinal clinical data. ConSequence further allows users to apply rule-based constraints to prevent the generation of patterns that never occur in the real data and to tailor outputs to specific requirements [17]. As language models, however, HALO and ConSequence represent all variables as discrete tokens. This means that continuous variables must be discretised prior to modelling, leading to some loss of granularity. Similarly, neither method directly models absolute timestamps (such as the date of an event), as they instead capture discretised time intervals between events. Although this preserves the temporal order of patient trajectories, it produces synthetic datasets whose structure and format differ from the original real data, limiting their compatibility with typical analytical workflows. In addition, previous evaluations have focused on two moderately-sized datasets (a large outpatient claims dataset and the MIMIC-III inpatient dataset). This leaves open questions about how such models perform at the extremes of dataset size, from small longitudinal studies to large-scale multi-hospital data covering millions of patients.

This study builds on the HALO and ConSequence frameworks by developing a pipeline for generating analysis-ready realistic synthetic longitudinal clinical data. The pipeline is applied across three datasets that differ in size, structure, and complexity, enabling a systematic examination of how synthetic data behaves in varied settings.

## The paper makes the following specific methodological contributions:

- reconstructs timestamps and continuous variables from discretised sequences, producing synthetic datasets aligned with real EHR table structures;
- supports conditional generation for targeted cohort synthesis;
- comprehensively evaluates realism and utility across machine learning, statistical modelling, and time series analysis using three clinical datasets of varying size and complexity; and
- provides practical guidance on when synthetic longitudinal data can be used effectively.

Taken together, these contributions address key barriers to adopting realistic longitudinal synthetic data in real clinical workflows.

## Methods

### Datasets

Three clinically diverse datasets were used to evaluate the synthetic data pipeline: the publicly available Alzheimer's Disease Neuroimaging Initiative (ADNI) dataset and the Medical Information Mart for Intensive Care (MIMIC-III) datasets, and an electronic health record dataset from the state of Queensland in Australia. As Table 1 shows, these datasets vary considerably in size, structure, and complexity.

Data used in the preparation of this article were obtained from the Alzheimer's Disease Neuroimaging Initiative database (adni.loni.usc.edu),. It is a multi-phase, longitudinal study that includes over 2,400 participants [18]. Although it is not an EHR dataset, it is included due to its longitudinal clinical structure and the distinct challenges it presents for synthetic data generation. The ADNI was launched in 2003 as a public-private partnership, led by Principal Investigator Michael W. Weiner, MD. It includes extensive clinical, neuroimaging, biomarker, and genetic data collected at regular follow-up intervals. The original goal of ADNI was to test whether serial magnetic resonance imaging (MRI), positron emission tomography (PET), other biological markers, and clinical and neuropsychological assessment can be combined to measure the progression of mild cognitive impairment (MCI) and early Alzheimer's disease (AD). The current goals include validating biomarkers for clinical trials, improving the generalizability of ADNI data by increasing diversity in the participant cohort, and to provide data concerning the diagnosis and progression of Alzheimer’s disease to the scientific community. For up-to-date information, see adni.loni.usc.edu

Data were also obtained from the MIMIC-III database [19], available via PhysioNet [20]. Access was granted after completion of the required data use agreement and human subjects training. MIMIC-III is a medium scale, publicly available ICU dataset with irregular visits and a complex schema including many separate tables [19].

The data from Queensland, Australia contains emergency department (ED) and inpatient encounters for 3.5 million patients over six years. It represents a very large-scale administrative dataset with highly heterogeneous patient trajectories and high-dimensional clinical information.

The variables and tables used from each dataset are listed in Table 2. These were selected to reflect common features in clinical datasets and to support the evaluation of synthetic data. For the MIMIC-III dataset, diagnosis and procedure ICD-9 codes were aggregated based on their first three characters. For the Queensland dataset, diagnosis ICD-10-AM codes occurring fewer than 50 times were aggregated to their first two or three characters to reduce sparsity. The maximum number of visits per patient was limited to 30 for ADNI, 48 for MIMIC-III, and 100 for Queensland data.

## Model architecture and training

### HALO and ConSequence implementation

The HALO model and ConSequence implementations were adapted from source code available at https://github.com/btheodorou/KnowledgeInfusion that accompanies the ConSequence publication [17]. Several modifications were made to simplify integration and improve reproducibility. These included replacing custom Conv1D layers with standard linear layers and dynamically generating causal masks at runtime. The forward pass was also modified to return raw logits rather than probabilities, allowing the use of BCEWithLogitsLoss for improved numerical stability during training. Optional mixed-precision support was also added for more efficient training and generation on modern GPUs. These adjustments make the model simpler to extend and reproduce, and they also enable compatibility with libraries such as Opacus. The models were implemented in Python 3.9 and PyTorch 1.13.

#### Model training

The model parameters used for each dataset are shown in Table 3. These were selected through experimentation guided by the aim of balancing model complexity with computational cost and the risk of overfitting. Where not specified, hyperparameters followed those reported in the original HALO and ConSequence papers. For the ADNI and MIMIC-III datasets, models were trained using two NVIDIA V100 GPUs, whereas the Queensland data model was trained using four NVIDIA H100 GPUs. 80% of the data was used for training, 10% for validation, and 10% for testing.

## Post-processing to produce EHR-formatted output

### Continuous variable reconstruction

To improve the realism of continuous variables in the synthetic data, a post-processing step based on kernel density estimation (KDE) was applied. For each continuous variable (e.g., age, time since previous visit), its empirical distribution was estimated using KDEMultivariate from the statsmodels package [21], stratifying by relevant covariates such as gender and event type. The cumulative distribution function (CDF) was then evaluated on a discretised grid of values and derived the probability mass associated with each grid interval. This allowed fine-grained empirical patterns to be captured.

For pairs of correlated continuous variables such as total length of stay and treatment length of stay, this approach was extended by computing joint interval probabilities from the joint CDF using the inclusion-exclusion principle.

To regenerate continuous variables from the categorical outputs of HALO and ConSequence, the pre-computed KDE-based probabilities were used in a two-stage sampling process. For each subgroup defined by categorical variables (e.g., gender = “Female”, age band = “30–35”), the KDE-derived probability mass for each finer interval inside the band was retrieved (e.g., 30–31, 31–32, etc.). One of these subintervals was then sampled according to its probability. Within the selected subinterval, a value was then drawn uniformly at random. This procedure maintained both the marginal distributions and key correlations observed in the original data while aligning with the categorical outputs of the generative models.

### Timestamp reconstruction

To reconstruct realistic event dates, patients were first stratified by the total number of visits and a plausible starting week was then sampled from a KDE-derived probability grid. This ensured that patients with different visit frequencies had appropriately distributed starting dates. For each visit, a day of the week was sampled from an empirical distribution, the number of weeks since the previous visit was calculated using the time since previous event variable, and then the cumulative event week was computed by summing the intervals and adding the first event week. The result was then mapped to a calendar date. This approach preserved both the temporal spacing between visits and the distribution of visit timing across the cohort.

For the MIMIC-III and Queensland datasets, a multi-tiered logic was implemented to regenerate realistic admissions and discharge timestamps from synthetic continuous variables (length of stay, inter-visit interval, and event timing features such as event week, event day of week, and event hour). The logic depended on the length of stay duration:

- For stays up to 24 hours long, discharge time was computed by directly adding the length of stay to the admission time.
- For stays between 1 and 7 days long, length of stay was converted to integer days and discharge time was adjusted using the synthetic end hour.
- For stays over 7 days long, length of stay was converted to weeks and discharge time was derived by mapping the synthetic event end week and end day to a calendar date using a lookup table and then adjusted with event end hour.

For subsequent admissions, admission type was calculated based on the previous discharge time and the inter-visit interval, with different logic applied depending on whether the interval was up to 24 hours, 1–7 days, or more than 7 days. The logic was similar to that used for deriving discharge time from admission time and length of stay.

## Diagnosis code reconstruction

For the Queensland dataset, there was an additional post-processing step for diagnosis codes. Each diagnosis code that had been collapsed into a higher-level two- or three-character category was mapped back to a specific ICD10 code by sampling from the empirical distribution of codes within that category stratified by gender. This procedure preserved the original spectrum of diagnosis codes in the final synthetic data, despite the use of a more compact code set during modelling.

### Synthetic data evaluation

This study evaluates realism and analytical utility; privacy risk assessment was not performed. All evaluation was conducted using R 4.0.

## Realism

Data was evaluated for realism through descriptive statistics, correlation matrices, diagnosis code frequencies, and measures of diagnosis code co-occurrence within and between visits (bigrams and conditional probabilities). Bigram probabilities for each unique pair of diagnosis codes within and between consecutive visits were calculated following a similar approach as in [15]. To assess preservation of clinically meaningful temporal relationship, conditional occurrences of clinically relevant diagnosis pairs were calculated for any later visit following the index visit.

## Machine learning utility

To assess machine learning performance, gradient-boosting models were trained for a classification task using the tidymodels R package [22]. For each of the three datasets, models were trained on real data, synthetic data, or a combination of both. Outcomes were selected that are commonly modelled in predictive analytics and mirror the types of tasks for which real data might be used. For ADNI, the classification task involved predicting whether a subject with no prior diagnosis of Alzheimer’s disease would receive a diagnosis at their next visit. For MIMIC-III, the task was to predict in-hospital mortality using only features available at the start of the ICU stay. For the Queensland dataset, the task involved predicting whether an ED visit will be followed by a subsequent ED visit within the next 30 days.

Machine learning performance was evaluated on a held-out test set of real data across three key dimensions: predictive performance, interpretability, and fairness. Predictive performance refers to how well a model can correctly predict outcome. This was assessed using standard classification metrics such as precision, recall, F1 score, PR AUC, and ROC AUC. Interpretability was assessed using SHAP (Shapley Additive exPlanations) values [23] to determine feature importance scores for each model, calculated using the shapviz package [24]. To quantify the consistency in feature importance between models trained on real and synthetic data, feature rankings were then compared using Spearman rank correlation. Fairness was evaluated using the equal opportunity metric [25].

## Statistical modelling utility

Performance in statistical modelling was evaluated by fitting statistical models to real and synthetic ADNI, MIMIC-III, and Queensland data. Model coefficients for models trained on real and synthetic data were then compared. For ADNI, a linear mixed-effects model was used to predict ADAS13 cognitive scores, incorporating fixed effects for demographic and genetic risk factors (e.g. age, education, APOE4 status) and random intercepts and slopes for time since baseline at the subject level. For MIMIC-III, ICU length of stay was modelled using a generalised linear model and included predictors such as age, gender, admission type, and clinical indicators (e.g. pneumonia, stroke). For the Queensland dataset, a linear mixed-effects model was used to predict ED length of stay, with fixed effects for triage category, arrival mode, and age, and a random intercept for facility. Additionally, the prevalence of six conditions (asthma, COPD, diabetes, myocardial infarction, stroke, and substance abuse) in the real and synthetic data was modelled using generalised additive models to examine how well synthetic data captures key epidemiological patterns.

## Time series analysis utility

Performance in time series analysis was evaluated using the Queensland dataset only, as this was the only dataset that included its original, unshifted timestamps and was expected to exhibit complex temporal patterns. A linear mixed-effects model was used to predict ED length of stay, with fixed effects for triage category, arrival mode, and age, and a random intercept for facility as well as for temporal variables such as month and day of week. Model coefficients for models trained on real and synthetic data were then compared. To further assess whether the synthetic data preserved aggregate temporal patterns, autocorrelation structure of daily ED presentations and inpatient admissions in the Queensland dataset were examined using ACF and PACF plots.

## Results

**Figure 1:**
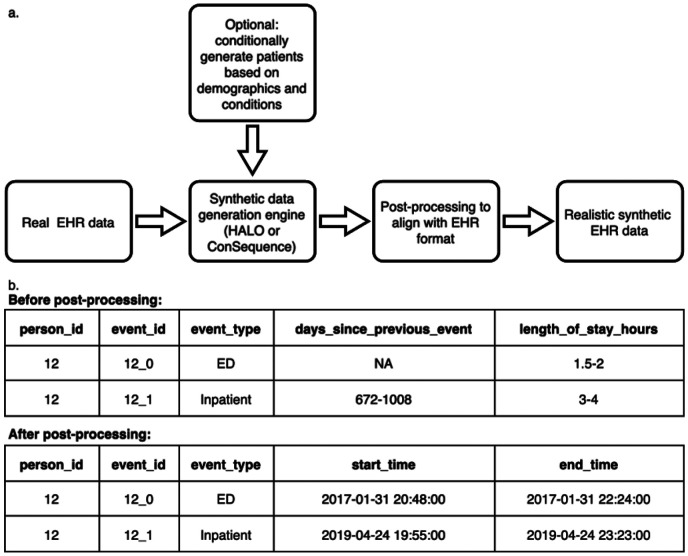
Overview of the synthetic data generation pipeline. a) The pipeline builds on the HALO or ConSequence model engine with a post-processing step and includes optional conditional generation. b) The post-processing step improves synthetic data format by adding timestamps

The synthetic data generation pipeline uses HALO or ConSequence as its core engine and is summarised in Fig. 1a. A key feature of the pipeline is the post-processing step which reconstructs timestamps and continuous variables (illustrated in Fig. 1b). This step produces realistic start and end times consistent with the original ranges and brings the synthetic output into alignment with the format of real data, substantially improving its suitability for downstream analyses such as machine learning. The pipeline also supports conditional generation based on specified patient demographics (e.g., gender, age at first visit) and clinical characteristics (e.g., conditions such as asthma, cancer, dementia, diabetes, heart disease, or stroke).

### The pipeline produces realistic synthetic data across clinically diverse datasets

To demonstrate its robustness, the synthetic data generation pipeline was applied to three clinically diverse datasets to capture a range of real-world challenges and use cases. Although HALO and ConSequence have been thoroughly validated in prior work [15, 17], the heterogeneity of the three datasets and changes made to the implementations warranted independent validation of the pipeline on each. As shown in Table 1, these datasets vary considerably in size, patient demographics, structure, and complexity. Each presents unique challenges, making this a strong test of the generalisability of the pipeline.

Table 4 summarises the distributions of key variables in the real and synthetic datasets. This includes patient age, gender, total visits per patient, days between visits, and length of stay. The distributions are generally similar between real and synthetic data, indicating that the synthetic data captures key characteristics of the original datasets. Notably, the close alignment in the distribution of days between visits in real and synthetic data suggests the method effectively preserves longitudinal structure.

**Figure 2:**
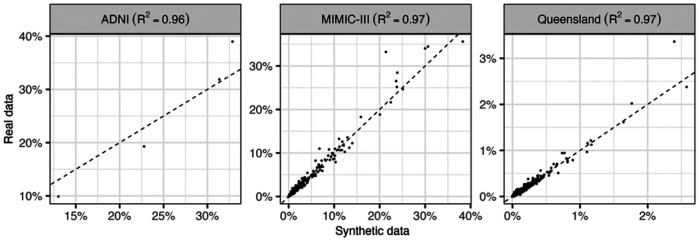
Frequencies of diagnosis codes in real and synthetic data

Additionally, the number of unique diagnosis codes was examined to see how well rare events are captured. Some synthetic data generation methods tend to underrepresent relatively rare codes, but the results show that the synthetic datasets retained at least 85% of diagnosis codes found in the real datasets. This indicates that the pipeline effectively captures even low frequency events. Figure 2 confirms that the frequencies of diagnosis codes in the synthetic data were very similar to those in real data, with R^2^ values of at least 0.96.

**Figure 3:**
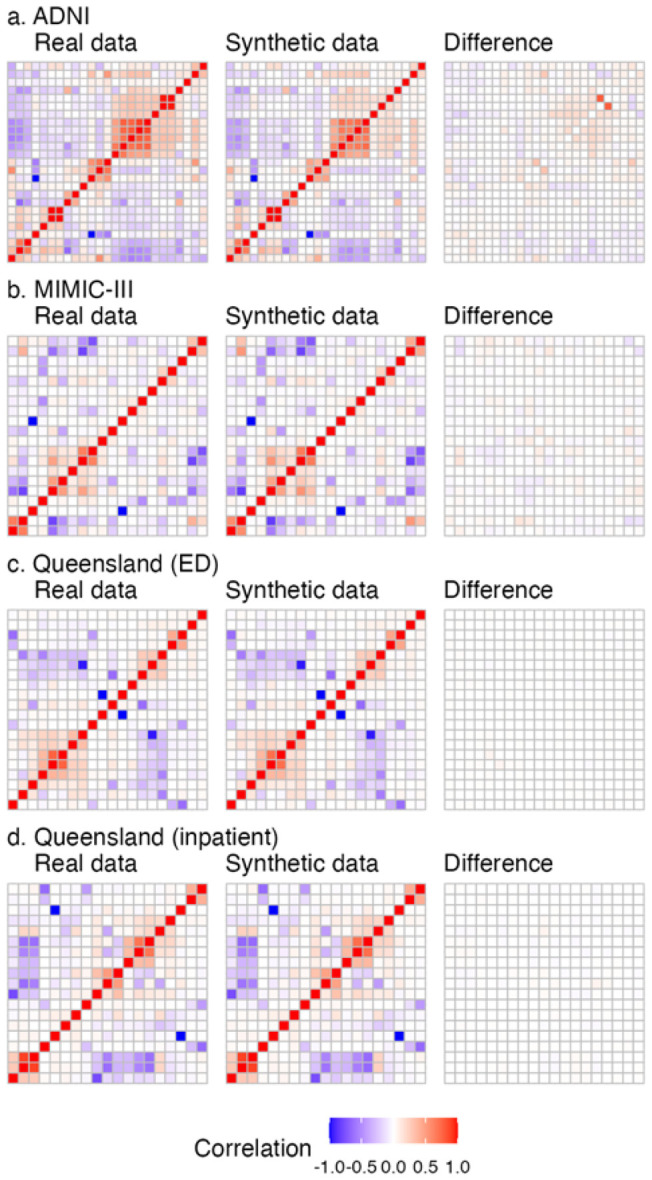
Correlation matrices for real data, synthetic data, and the difference between them for the a) ADNI, b) MIMIC-III, c) Queensland (ED), and d) Queensland (inpatient) datasets

As well as capturing the distributions of individual variables, high quality synthetic data should also preserve the relationships between variables. To assess this, pairwise correlations between key variables in the real and synthetic datasets were compared. Figure 3 shows the correlation matrices for real data, synthetic data, and the corresponding difference matrix for each dataset. Note that for the Queensland datasets ED and inpatient visits are shown separately, as there are many variables that are specific to either ED or inpatient visits.

In each real dataset there are many highly correlated variables, indicating that there are complex multivariate relationships that are important to preserve in the synthetic data. The correlation patterns in the synthetic data closely match those in the real data, with only minor deviations. The largest deviations seen are in the ADNI dataset, which is also the smallest, while the very large Queensland dataset shows the closest alignment. This suggests the pipeline is able to capture complex relationship between variables well even for small datasets, though larger datasets support higher fidelity.

Realistic synthetic EHR data must not only capture distributions of variables and the relationships between them, but also clinical trajectories seen in real patient data. This is one of the major challenges of modelling longitudinal data, as each visit must be coherent within the context of the patient’s overall clinical progression. Two separate approaches to evaluate whether our synthetic data preserves realistic clinical trajectories.

Firstly, the bigram probabilities for each pair of diagnosis codes in the same visit and in consecutive visits were compared using a similar approach as in [15]. The R^2^ values for these probabilities are shown in Table 5. For all three datasets, the correlations are very high showing that the synthetic data effectively captures patterns of diagnosis progression seen in the real data, as well as co-occurrences of diagnosis codes within a visit.

**Figure 4:**
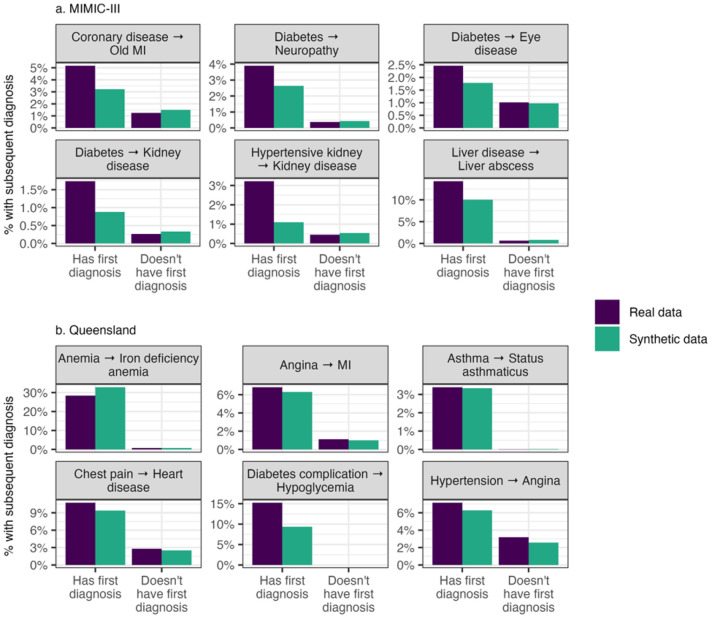
Conditional occurrence of subsequent diagnoses after an index diagnosis in the a) MIMIC-III and b) Queensland datasets

Secondly, conditional occurrences of specific diagnoses following an index diagnosis were examined for the MIMIC-III and Queensland datasets. Whereas the previous approach considered only consecutive visits, here diagnoses in any visit following the index visit were included. This captures potential longer term clinical progressions that may not be apparent over two consecutive visits. These results are shown in Fig. 4.

For both datasets, the synthetic data accurately captured the increased prevalence of specific diagnoses among patients with a history of an index diagnosis. For example, in the MIMIC-III dataset, patients with a prior diagnosis of diabetes were more likely to subsequently develop conditions such as eye disease, kidney disease, and neuropathy. While the magnitude of these increased risks was sometimes slightly lower in the synthetic data, the overall pattern of elevated risk was well preserved.

Similarly, in the Queensland dataset we see that the increased prevalence of a subsequent diagnoses following specific index diagnoses was captured well. For instance, hypoglycaemia was diagnosed in approximately 15% of patients who had previously experienced a diabetes-related complication but was very rare among other patients. In the synthetic data, this elevated risk was slightly attenuated, with hypoglycaemia occurring in approximately 10% of patients with prior diabetes complications. Nevertheless, the diagnosis was extremely rare in the rest of the population, suggesting that the synthetic data preserved the overall clinical pattern well.

### Conditional generation produces realistic patient cohorts

HALO and ConSequence both have the capability for conditional generation [15]; that is, to specifically generate patients with specific diagnoses, demographics, or other characteristics. This is accomplished by including indicator variables in the patient labels that are then used during synthetic data. To test this capability, patients with one of six diagnoses (asthma, cancer, dementia, diabetes, heart disease, and stroke) were conditionally generated using the Queensland dataset. Table 6 shows the prevalence of each diagnosis in the conditionally generated synthetic cohort, unconditioned synthetic data, and real data. In the synthetic cohorts, between 77 and 97% of patients actually have the relevant diagnosis code, which represents a significant enrichment compared to both unconditioned synthetic data and real data.

**Figure 5:**
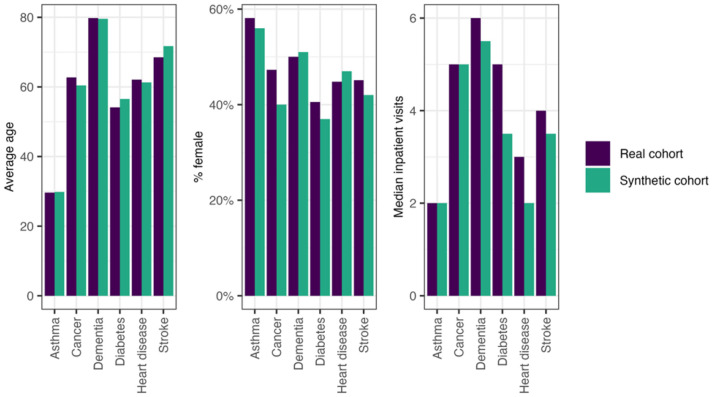
Average age, percentage of female patients, and median number of Queensland inpatient visits for conditionally generated cohorts with specific diagnoses

For these conditionally generated cohorts to be useful in downstream applications, diagnoses need to be embedded within plausible patient profiles rather than simply being assigned in isolation. Figure 5 compares the average age, proportion of female patients, and median number of inpatient visits for each synthetic cohort and patients with the corresponding diagnosis in the real data. The similarities in age distribution, gender balance, and healthcare utilisation indicate that the diagnosis-specific synthetic cohorts capture realistic patient characteristics. For instance, the synthetic dementia cohort has an average age of around 80 years, whereas the asthma cohort is much younger. This mirrors the expected demographic patterns observed in the real data.

### Synthetic data supports a range of downstream applications

The utility of synthetic data was also evaluated across a range of downstream tasks commonly encountered in clinical and health data science, including machine learning, statistical modelling, and time series analysis. The aim of this was to establish when synthetic data can reliably replace or augment real data. It is important to understand the limitations of synthetic data and any conditions where it is not appropriate to use it in place of real data.

To assess machine learning performance, gradient-boosting models were trained for a classification task. For each of the three datasets, models were trained using real data, synthetic data, or a combination of both. Outcomes were selected that are commonly modelled in predictive analytics and mirror the types of tasks for which real data might be used. Machine learning performance was assessed across three key dimensions: predictive performance (Table 7), interpretability (Table 8), and fairness (Table 9).

Table 7 shows that for the two larger datasets (MIMIC-III and Queensland data), the predictive performance of models trained on synthetic data is very similar to that of models trained on real data. In contrast, for the much smaller ADNI dataset the model trained on synthetic data performed worse. Combining real and synthetic data yielded the best performance for ADNI, whereas for the two other datasets no performance boost was seen when augmenting real data with synthetic data.

Table 8 shows the top ten features identified for machine learning models trained on either real or synthetic data for each of the three datasets. For each of the three datasets, at least eight of the top ten features are shared in models trained on real and synthetic data, and the overall correlations range from 0.65 to 0.97. This indicates strong agreement in feature importance between models trained on real and synthetic data.

Table 9 shows equal opportunity scores for machine learning models trained on real, synthetic, and combined data for ADNI, MIMIC-III, and Queensland data. For each dataset, equal opportunity scores were calculated for at least two variables such as age, gender, ethnicity, or IRSAD (Index of Relative Socio-economic Advantage and Disadvantage), depending on which variables were present in each dataset. For the Queensland dataset, equal opportunity scores were very similar across all three training conditions. In MIMIC-III, synthetic data produced slightly larger disparities than real data, but augmenting real data with synthetic data reduced these differences and yielded scores comparable to or better than real data alone. In contrast, for ADNI, models trained on synthetic or combined data showed noticeably greater disparities than those trained solely on the real dataset. Taken together, these results suggest that dataset size may influence how well fairness properties are preserved in synthetic data.

**Figure 6:**
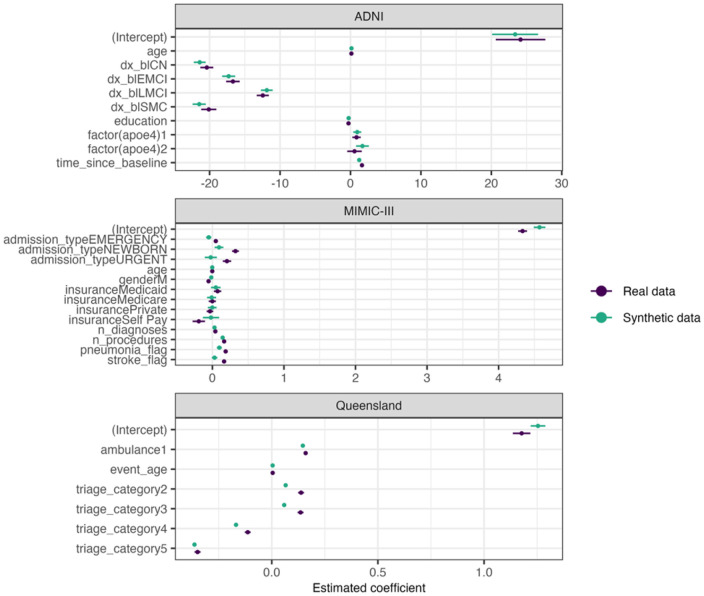
Coefficient estimates and confidence intervals from statistical models trained on real and synthetic data across the ADNI, MIMIC-III, and Queensland datasets

To explore how well synthetic data performs in statistical modelling, models were fitted to real and synthetic ADNI, MIMIC-III, and Queensland data. Coefficients for models trained on real and synthetic data were then compared to assess whether synthetic data preserves key patterns and associations (Fig. 6). Overall the coefficients derived from synthetic data were similar to those from real data, indicating that key relationships were largely captured accurately. While confidence intervals did not always overlap, the direction and relative magnitude of effects were consistent. For example, in the ADNI dataset the estimates for baseline diagnosis variables (prefixed with dx_bl) differed slightly for real and synthetic data. However, the ordering (SMC and CN being lowest, followed by EMCI and then LMCI) was maintained. Similar consistency was observed in the MIMIC-III and Queensland datasets.

**Figure 7:**
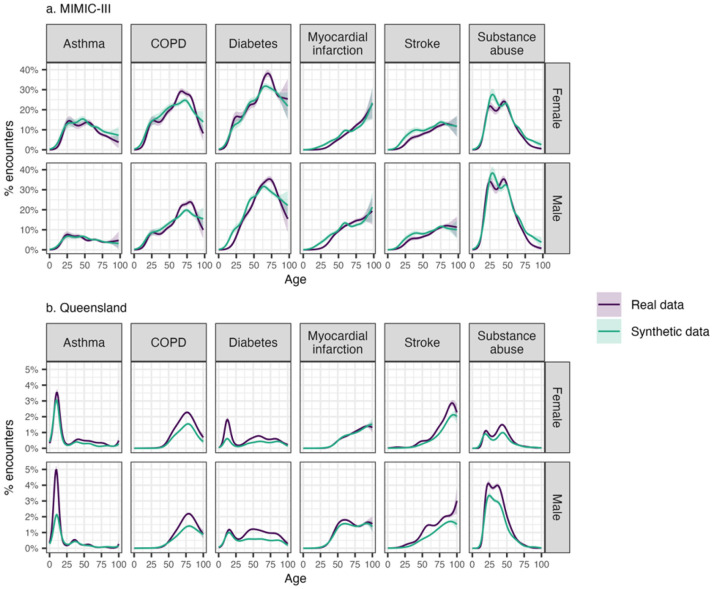
Modelled prevalence of six conditions across gender and age groups in the MIMIC-III and Queensland datasets, comparing outputs from models trained on real and synthetic data

Disease prevalence by gender and age were also modelled to evaluate how well synthetic data captured key epidemiological patterns (Fig. 7). In general, synthetic data captured the key patterns observed in the real data accurately. For example, the age-related increases in conditions like COPD, myocardial infarction, and stroke were preserved, as were gender differences in substance abuse. However, some discrepancies in magnitude were evident. For example, while the synthetic Queensland data preserved the overall pattern of higher asthma prevalence in those under 20, it underestimated the prevalence for males in this age group compared to the real data.

Finally, the utility of synthetic data for time series analysis was also evaluated. Although the post-processing step in the synthetic data pipeline restores timestamps to records, this does not guarantee that overall temporal patterns are accurately reproduced. As a result, it is essential to evaluate whether synthetic data preserves meaningful aggregate temporal patterns.

**Figure 8:**
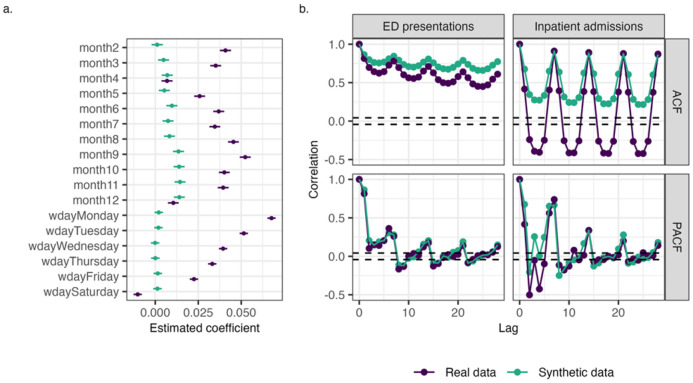
a) Coefficient estimates for temporal variables from statistical models trained on real and synthetic Queensland data b) Comparison of autocorrelation (ACF) and partial autocorrelation (PACF) characteristics for daily ED presentations and inpatient admissions in real and synthetic Queensland data

Figure 8a shows the coefficient estimates for temporal variables from statistical models trained on real and synthetic Queensland data. This model builds on the one shown in Fig. 6, with the addition of temporal variables such as month and day of the week. For clarity, only the time variables are shown. While Fig. 6 showed strong agreement between real and synthetic data for non-temporal variables, for temporal variables the coefficient estimates differ substantially. Notably, the clear yearly and weekly patterns seen in the real data are not preserved in the synthetic data.

To further assess whether synthetic data preserves aggregate temporal patterns, the autocorrelation structure of daily ED presentations and inpatient admissions in the Queensland dataset were also examined using autocorrelation (ACF) and partial autocorrelation (PACF) plots (Fig. 8b). This approach assesses a different aspect temporal fidelity than the comparison of model coefficients above. While coefficient comparisons can reveal whether synthetic data reproduces expected calendar effects, autocorrelation analysis assesses the persistence of values over time. Examining both ACF and PACF is important because the capture different aspects of temporal structure. ACF reflects broader temporal trends that emerge from the aggregation of many individual records over time, while PACF isolates direct relationships at specific lags. In the ACF plots, the real data shows clear peaks at 7-day intervals, consistent with weekly patterns in healthcare utilisation. However, these are substantially dampened or absent in the synthetic data, indicating that the aggregate temporal dependencies weren’t captured well. In contrast, the PACF plots appear more similar between real and synthetic data, suggesting that synthetic data may preserve short-term autoregressive structure (e.g., correlations within a few days) while failing to reproduce longer term or cumulative dependencies.

## Discussion

This study develops and comprehensively evaluates a realistic synthetic data generation pipeline built on the state-of-the-art HALO and ConSequence models [15, 17]. The realism and utility of generated datasets are examined across diverse clinical datasets and analytical tasks. Overall, the pipeline produces realistic and analytically useful synthetic data across a range of dataset sizes, structures, and complexities. By reconstructing timestamps and continuous variables through a post-processing step, the pipeline also aligns the synthetic data format with that of real data to improve usability in downstream applications. This addresses a limitation of the HALO framework. Although in the original HALO publication the authors show continuous values can be reconstructed through uniform sampling within discretised buckets [15], this does not capture the empirical shape of the underlying distribution. Our pipeline therefore increases realism in both marginal distributions and joint relationships.

The results show that the synthetic data closely matches real data in terms of variable distributions, inter-variable relationships, and clinical trajectories. The largest deviations seen were in the ADNI dataset, which is also the smallest, while the very large Queensland dataset showed the closest alignment. This suggests that the fidelity of synthetic data is constrained by the amount of real data available for training, with larger datasets generally supporting higher fidelity.

The ability to conditionally generate patients with specific diagnoses was also explored. This capability is particularly useful for applications that require enrichment of small or under-represented cohorts. In each disease cohort at least 77% of patients actually had the target diagnosis. Because the underlying model engines are probabilistic, perfect concordance is not expected. Nonetheless, this represents a very substantial enrichment compared with unconditioned synthetic data. Importantly, these cohorts were clinically coherent, with patients also displaying characteristics commonly associated with the target condition in the real data.

One practical use of this capability is subgroup rebalancing: generating additional records for combinations of protected attributes and outcomes that are underrepresented in the real data. In recent work, Theodorou et al. [26] use HALO in this way to equalise group and label frequencies before downstream model training, improving both overall performance and subgroup fairness without modifying model architectures or loss functions. This demonstrates the utility of conditional generation for targeted data augmentation.

Although fidelity is a promising indicator of utility, this study explicitly evaluated the utility of synthetic data for several applications to better understand when synthetic data can be used in place of real data and where it has limitations. Utility was assessed for three common health data science applications: machine learning, statistical modelling, and time series analysis.

Machine learning models trained on synthetic data had comparable accuracy to those trained on real data, especially for the two larger datasets. Interestingly, for the ADNI dataset the best performance was observed when real data was augmented with synthetic data. This suggests that combining real data with high-quality synthetic data may be beneficial when working with small datasets. However, this performance boost was not seen for the other two datasets, indicating data augmentation is of less benefit for larger datasets.

Importantly, interpretability and fairness were assessed alongside predictive performance. For health applications, interpretability is critical because clinicians and regulators must be able to understand how predictions are made [27]. Although a model trained on synthetic data may match the predictive accuracy of one trained on real data, differences in its underlying decision process could undermine its clinical utility. Similarly, fairness is critical to assess, as biases present in real data could be amplified in synthetic data and lead to disparities of care.

Feature importance rankings were highly consistent between models trained on real and synthetic data, suggesting that the models depend on similar features to make predictions and thus would similarly align with clinicians’ expectations. Fairness was also examined, as prior work has shown that synthetic healthcare data can amplify biases in real data leading to different fairness behaviour in downstream models [28]. Fairness analyses showed broadly similar, and in some cases slightly larger, disparities for models trained on synthetic data, with more pronounced differences in the relatively small ADNI dataset. Overall, larger datasets appeared to yield better preservation of fairness properties. Importantly, substantial disparities were also observed in some models trained purely on real data, reinforcing that this is not a unique failure mode of synthetic data.

Statistical modelling is arguably a more stringent test of synthetic data utility than machine learning. While machine learning models are optimised for predictive performance and are often black boxes, statistical models are used to understand and quantify relationships between variables. Their coefficients are used to estimate associations and causal effects, so they are particularly sensitive to the underlying data. This makes statistical modelling a crucial benchmark, as it tests whether synthetic data can support inference and explanation.

Synthetic data generally performed well in statistical modelling. In almost every case coefficients that were statistically significant in models trained on real data remained significant in their synthetic counterparts, suggesting that synthetic data can support valid inference. However, if exact coefficient values are required (for example, in highly sensitive risk prediction or causal inference tasks) synthetic data may not be suitable. Similarly, patterns in disease prevalence by gender and age were overall captured well, though in some cases there were discrepancies in magnitude. This will likely be of greater concern for relatively rare conditions. These results suggest that while synthetic data can effectively support statistical inference by retaining essential structural patterns, its utility depends on the precision requirements of the specific application.

Our findings suggest that synthetic data produced using the pipeline is not suitable for use in applications reliant on population-level temporal patterns. This is not surprising given that the underlying model engine is optimised for capturing longitudinal dynamics at the individual level (such as disease trajectories), not broader population-level temporal trends. Although the post-processing step reconstructs timestamps, this step aims to simply align the format of the synthetic data with the real data rather than capture aggregate temporal patterns. Synthetic data generated using this approach is therefore not appropriate for modelling tasks that rely on aggregate time-based patterns, such as forecasting or seasonal trend detection.

A limitation of this study is that it did not evaluate privacy of the synthetic data. Although the HALO paper presents promising empirical evidence regarding privacy risks [15], neither HALO nor ConSequence provides differential privacy guarantees or mechanisms specifically designed to prevent memorisation of individual records. Because the focus in this work was on realism and analytical utility across diverse datasets, privacy evaluation was considered out of scope but is an important direction for future research.

This study does not repeat the baseline benchmarking performed in the original HALO and ConSequence papers. HALO has already been evaluated against the main families of synthetic EHR generators and demonstrated state-of-the-art performance across statistical fidelity, temporal modelling, predictive utility, and privacy evaluation [15]. Because the present work builds on HALO and ConSequence rather than proposing a new generative model, re-running those baselines would offer limited additional insight. The focus is instead on developing and evaluating a generalisable pipeline for producing realistic synthetic datasets suitable for downstream analytical tasks.

## Conclusion

This study evaluated a synthetic data generation pipeline based on the HALO and ConSequence models across three clinically diverse datasets that varied markedly in size, structure, and complexity. By incorporating a post-processing step to reconstruct timestamps and continuous variables, the pipeline produces synthetic data that more closely match the structure of real EHRs and is easier to use in downstream analyses. Across all datasets, the synthetic data were realistic and supported both statistical modelling and machine learning applications. However, despite preserving individual-level temporal information, the generated data were not suitable for analysis requiring population-level temporal patterns. Overall, the findings demonstrate the robustness of the approach across heterogeneous datasets, including relatively small cohorts, while highlighting limitations that should guide practical use and inform future improvements.

## Figures and Tables

**Table 1:** Overview of datasets used for synthetic data generation

Dataset	ADNI	MIMIC-III	Queensland data
No. patients	2409	46,520	3,536,423
Dataset type	Longitudinal study	ICU stays	Emergency and inpatient encounters
Visits per patient	1–18 (median: 3)	1–42 (median: 1)	1-1260 (median: 2)
Visit spacing	Regular	Irregular	Irregular
Timestamps	Real	Shifted for privacy (intervals preserved)	Real
Challenges	Rich dataset with relatively small cohort size Highly correlated variables Missing values	Many linked tables Loss of original timestamps	Very high volume Complex patient trajectories

**Table 2:** Summary of variables included from each dataset

Dataset	Table type	Variables
ADNI	Subjects	Gender, age, education, race, APOE4, baseline diagnosis
	Visits	Date, ADAS11, ADAS13, CDRSB, FAQ, hippocampus, ventricles, whole brain, ICV, TAU, PTAU
MIMIC-III	Patients	Gender, age, ethnicity
	Visits	Admission time, discharge time, admission type, admission location, hospital expire flag, insurance, length of stay
	Diagnoses	Diagnosis ICD-9 code
	Procedures	Procedure ICD-9 code
Queensland data	Patients	Gender, age, binary flags for six diagnosis-based condition groups (asthma, cancer, dementia, diabetes, heart disease, stroke)
	Visits	Start time, end time, event type, facility, IRSAD, remoteness, arrival mode, end status, visit type, triage category, elective status, episode type, separation mode, original reference code, clinical lead at discharge, DRG, unit, triage length of stay, treatment length of stay, departure length of stay, total length of stay
	Diagnoses	Diagnosis ICD-10-AM code

**Table 3:** Model parameters for each dataset

Dataset	Model	Layers	Batch size	Embedding dimension	Vocabulary size
ADNI	ConSequence	4	6	256	117
MIMIC-III	HALO	12	32	768	1689
Queensland data	HALO	12	256	768	7636

**Table 4:** Summary statistics for real and synthetic ADNI, MIMIC-III, and Queensland datasets

Dataset	Patient age (mean and s.d.)	% patients female	Visits per patients (median, IQR, range)	Days between visits (median, IQR, range)	LOS in hours (median, IQR, range)	Unique diagnoses
ADNI (real)	73 (7.3)	47.7%	4 [3–7], 1–18	350 [181–382], 10–1912	NA	3
ADNI (synthetic)	72.7 (7.2)	44.7%	4 [3–7], 1–18	348 [180–392], 1–1804	NA	3
MIMIC–III (real)	51.3 (28.2)	43.0%	1 [1–1], 1–42	120 [26–489], 0–4108	158 [91–286], 0–4954	936
MIMIC–III (synthetic)	51.3 (28.6)	44.8%	1 [1–1], 1–22	122 [29–467], 0–4108	129 [81–215], 0–4962	873
Queensland data (real)	36.4 (25.2)	50.6%	2 [1–5], 1–100	ED: 91 [13–313], −2–2187 Inpatient: 0 [0–19], −2–2178	ED: 3 [2–5], 0–235 Inpatient: 10 [3–51], 0–8760	11,784
Queensland data (synthetic)	36.2 (25.2)	48.1%	2 [1–5], 1–100	ED: 92 [12–318], −2–2161 Inpatient: 0 [0–15], −2–2153	ED: 3 [2–5], 0–277 Inpatient: 12 [3–51], 0-8807	10,095

**Table 5:** R^2^ values for diagnosis code bigram probabilities within the same visit and in sequential visits

Dataset	Same visit diagnosis codes	Sequential visit diagnosis codes
ADNI	NA	0.969
MIMIC-III	0.932	0.942
Queensland data	0.910	0.972

**Table 6:** Prevalence of selected diagnoses in conditionally generated synthetic cohorts compared with unconditioned synthetic data and real data

Condition	Prevalence in synthetic cohort	Prevalence in unconditioned synthetic data	Prevalence in real data
Asthma	96%	1.0%	1.3%
Cancer	93%	4.1%	4.6%
Dementia	77%	0.2%	0.4%
Diabetes	80%	0.5%	0.8%
Heart disease	97%	7.0%	7.8%
Stroke	85%	1.1%	1.4%

**Table 7:** Predictive performance of machine learning models trained on real, synthetic, or combined data across the ADNI, MIMIC-III, and Queensland datasets. The best value for each dataset and metric is shown in bold

Dataset	Training data	Precision	Recall	F1 score	PR AUC	ROC AUC
ADNI (positive class prevalence: 10%)	Real	0.49	0.65	0.56	0.52	0.92
	Synthetic	0.41	0.71	0.52	0.40	0.89
	Real + synthetic	**0.55**	**0.74**	**0.63**	**0.55**	**0.93**
MIMIC-III (positive class prevalence: 11%)	Real	0.21	**0.77**	0.33	0.26	0.77
	Synthetic	**0.23**	0.53	0.32	0.24	0.75
	Real + synthetic	0.22	0.75	**0.34**	**0.27**	**0.77**
Queensland data (positive class prevalence: 21%)	Real	**0.36**	0.58	**0.44**	0.45	**0.71**
	Synthetic	0.34	0.60	0.44	0.45	0.71
	Real + synthetic	0.34	**0.61**	0.44	**0.45**	0.71

**Table 8:** Top ten features ranked by SHAP values for machine learning models trained on real and synthetic data across the ADNI, MIMIC-III, and Queensland datasets. The overall Spearman rank correlation between rankings for all features is shown in the header, and features not shared across both models are italicised

	ADNI Overall correlation: 0.65		MIMIC-III Overall Correlation: 0.73		Queensland data Overall correlation: 0.97	
Feature rank	Real data	Synthetic data	Real data	Synthetic data	Real data	Synthetic data
1	No. MCI diagnoses	No. MCI diagnoses	Admission type	Admission location	Days since previous ED event	Days since previous ED event
2	ADAS13	ADAS13	Age	Admission type	Previous LOS	Facility
3	FAQ	Years since first visit	Admission location	Insurance	No. ED events within 180 days	Previous LOS
4	CDRSB	FAQ	Insurance	*Age over 70*	Days since previous inpatient event	Days since previous inpatient event
5	Years since first visit	No. CN diagnoses	Ethnicity	Age	Facility	No. ED events within 180 days
6	No. CN diagnoses	CDRSB	*Previous LOS*	Cumulative radiology and rehab procedures	S diagnosis	S diagnosis
7	*CN diagnosis*	FAQ rate	Cumulative radiology and rehab procedures	Ethnicity	End status	End status
8	FAQ rate	Hippocampus (normalised)	Organ deterioration	*Age over 80*	LOS	LOS
9	*ADAS13 rate*	*CDRSB rate*	*Age over 60*	Organ deterioration	No. ED events within 90 days	No. ED events within 90 days
10	Hippocampus (normalised)	*LMCI baseline diagnosis*	Time since previous stay	Time since previous stay	Triage category	Triage category

**Table 9:** Equal opportunity scores for machine learning models trained on real, synthetic, and combined data across the ADNI, MIMIC-III, and Queensland datasets. The best value for each dataset and variable is shown in bold

Dataset	Subgroup variable	Real	Synthetic	Real + Synthetic
ADNI	Age group	**0.24**	0.35	0.35
	Gender	**0.01**	0.17	0.12
MIMIC-III	Age group	0.66	0.74	**0.65**
	Gender	**0.04**	0.10	0.06
	Ethnicity	0.49	0.60	**0.46**
Queensland data	Age group	**0.23**	0.26	0.24
	Gender	0.01	**0.01**	0.01
	IRSAD	0.21	0.20	**0.20**

## Abbreviations

ACF: Autocorrelation function
ADNI: Alzheimer’s Disease Neuroimaging Initiative
ED: Emergency department
EHR: Electronic health record
ICD: International Classification of Diseases
IQR: Interquartile range
IRSAD: Index of Relative Socio-economic Advantage and Disadvantage
KDE: Kernel density estimation
LOS: Length of stay
MIMIC: Medical Information Mart for Intensive Care
PACF: Partial autocorrelation function
SHAP: Shapley additive explanations
